# Methylseleninic Acid Enhances Paclitaxel Efficacy for the Treatment of Triple-Negative Breast Cancer

**DOI:** 10.1371/journal.pone.0031539

**Published:** 2012-02-14

**Authors:** Yanfeng Qi, Xueqi Fu, Zhenggang Xiong, Haitao Zhang, Steven M. Hill, Brian G. Rowan, Yan Dong

**Affiliations:** 1 Departments of Structural and Cellular Biology, Tulane Cancer Center, Tulane University School of Medicine, New Orleans, Louisiana, United States of America; 2 College of Life Sciences, Jilin University, Changchun, Jilin, China; 3 Department of Pathology and Laboratory Medicine, Tulane Cancer Center, Tulane University School of Medicine, New Orleans, Louisiana, United States of America; 4 National Engineering Laboratory for AIDS Vaccine, College of Life Sciences, Jilin University, Changchun, Jilin, China; University of Medicine and Dentistry of New Jersey, United States of America

## Abstract

A major challenge in breast cancer therapy is the lack of an effective therapeutic option for a particularly aggressive subtype of breast cancer, triple-negative breast cancer. Here we provide the first preclinical evidence that a second-generation selenium compound, methylseleninic acid, significantly enhances the anticancer efficacy of paclitaxel in triple-negative breast cancer. Through combination-index value calculation, we demonstrated that methylseleninic acid synergistically enhanced the growth inhibitory effect of paclitaxel in triple-negative breast cancer cells. The synergism was attributable to more pronounced induction of caspase-mediated apoptosis, arrest of cell cycle progression at the G2/M checkpoint, and inhibition of cell proliferation. Treatment of SCID mice bearing MDA-MB-231 triple-negative breast cancer xenografts for four weeks with methylseleninic acid (4.5 mg/kg/day, orally) and paclitaxel (10 mg/kg/week, through intraperitoneal injection) resulted in a more pronounced inhibition of tumor growth compared with either agent alone. The attenuated tumor growth correlated with a decrease in tumor cell proliferation and an induction of apoptosis. The *in vivo* study also indicated the safety of using methylseleninic acid in the combination regime. Our findings thus provide strong justification for the further development of methylseleninic acid and paclitaxel combination therapy for the treatment of triple-negative breast cancer.

## Introduction

Triple-negative breast cancer (TNBC) refers to a subtype of breast cancer that is negative for expression of estrogen receptor and progesterone receptor, and lacks HER2 overexpression. The majority of TNBCs bear the gene expression profile of basal-like phenotype [1]. This subtype accounts for ∼10–15% of all types of breast cancer. TNBC is particularly aggressive, associated with rapid relapse following therapy, increased prevalence of distant metastases, and shorter survival comparing with other breast cancer subtypes [2]. It is more prevalent in Hispanic and African American women than in other ethnic groups, with the majority of the cases occurring in premenopausal women [3], [4].

Due to the “triple-negative” nature, patients with TNBC are not candidates for endocrine therapy or targeted therapy against HER2. TNBC patients are usually managed with standard treatments, and chemotherapy is the primary, if not the only, choice of systemic therapy. Taxanes, such as paclitaxel, alone or in combination with anthracycline reagents and/or alkylation reagents are the first-line chemotherapeutic drugs being used for TNBC [2], [5]. Although patients generally have a favorable initial response to taxane regimens, rapid development of resistance to taxanes is prevalent [5]. Therefore, developing agents that could effectively enhance the efficacy of taxanes and overcome resistance is urgently needed for the management of TNBC.

Selenium is an effective anticancer agent with a low toxicity profile [2]. The anticancer efficacy depends on the form and dosage of selenium administered [6]–[11]. Methylseleninic acid (MSA) is a potent second-generation selenium compound. It has very different biological and pharmacological activity than selenomethionine, the form of selenium used in the Selenium and Vitamin E Chemoprevention Trial [7], [9]–[13]. MSA has been shown to enhance the efficacy of chemotherapeutic drugs in cultured estrogen-receptor-positive breast cancer cells as well as cultured prostate cancer cells and prostate tumor xenografts [14]–[17]. However, the effectiveness of MSA alone or in a combination regime for treating TNBC has never been evaluated. In this report, we describe a series of experiments that were designed to determine the potential of using MSA to improve the therapeutic outcome of paclitaxel for the treatment of TNBC.

## Materials and Methods

### TNBC Cell Lines and Reagents

MDA-MB-231, MDA-MB-157, and BT-549 cell lines were obtained from American Type Culture Collection. MDA-MB-231 and MDA-MB-157 cells were cultured in DMEM medium, and BT-549 cells in RPMI 1640 medium. Both media were supplemented with 10% fetal bovine serum, 2 mM glutamine, 100 units per ml penicillin, and 100 µg/ml streptomycin. Paclitaxel and MSA were purchased from Sigma.

### Cell viability, Apoptosis, and Cell Proliferation Detection

Cell viability was determined by using either the Sulforhodamine B (SRB) assay as described [18] or direct cell counting with trypan blue staining. Cell proliferation was measured by using the BrdU Cell Proliferation ELISA kit (Roche), and apoptosis of cultured cells detected by using the Cell Death Detection ELISA^PLUS^ Kit (Roche) as described [19], [20]. The apoptotic cells on tumor sections were detected by the TUNEL assay using the *In Situ* Cell Death Detection Kit-TMR red (Roche) per manufacturer's protocol. The TUNEL slides were counterstained with DAPI for nuclear staining. The images were taken by using a Nikon Eclipse 80i fluorescence microscope, and quantitated with the use of the Image J software.

### Western blot analysis

The procedure was described previously [21]. Immune-reactive bands were quantitated by densitometry using the Licor Odyssey software and normalized to glyceraldehyde-3-phosphate dehydrogenase (GAPDH). The antibodies for the active forms of caspase-3 (Catalog Number 9664) and caspase-7 (Catalog Number 9491), and cleaved PARP (Catalog Number 5625) were obtained from Cell Signaling. The GAPDH antibody was from Millipore (Catalog Number Mab374).

### Cell Cycle Analysis

Cells were detached from culture dishes by using 15 mM EDTA-Na_2_, and then fixed in 70% ethanol. The cell pellet was washed with PBS twice and suspended in propidium iodide staining solution (0.1% (v/v) Triton X-100, 0.2 mg/ml DNase-free RNase A, and 20 µg/ml propidium iodide). After incubation at room temperature for 30 min, DNA content was analyzed by using the BD LSRII flow cytometer. The Modfit LT™ software was used to analyze the data.

### Caspase-3/7 Activity Assay

Caspase-3/7 activity was measured by using the Caspase 3/7-Glo assay kit (Promega) according to manufacturer's manual. The reading was measured by using the Fluostar Optima fluorescent plate reader.

### MDA-MB-231 Tumor Xenograft Model

Female SCID hairless out-bred mice were obtained from Charles River at 8 weeks of age. The mice were kept in vivarium for two weeks to adapt before any procedure. One million MDA-MB-231 cells in PBS were inoculated into both sides of the mammary fat pad with Matrigel. When tumors were palpable, ∼4–6 weeks after the inoculation, the mice were randomly assigned to four groups. Control mice were given weekly intraperitoneal saline solution. The MSA group was treated with 3 mg selenium in the form of MSA per kg body weight daily by an oral route [22]. Paclitaxel, 10 mg/kg, was given by intraperitoneal injection weekly. The combination group was treated with daily oral MSA and weekly intraperitoneal paclitaxel. Body weights and tumor dimensions were monitored weekly. Tumor volume was calculated as *0.524 x width^2^ x length*. At the end of the experiment, mice were anesthetized, and tumors removed, weighed, and fixed in 10% formalin for paraffin embedding and histological analyses. All animal procedures were approved by the Tulane University Institutional Animal Care and Use Committee.

### Immunohistochemical (IHC) Analysis

Paraffin-embedded blocks were sectioned, deparaffinized, rehydrated, and quenched by immersing in 1% H_2_O_2_. The slides were boiled in 10 mM citrate buffer (pH 6.0) for 20 min for antigen retrieval, and pre-incubated with 5% blocking serum to prevent non-specific binding. Ki-67 antibody incubation was applied at room temperature for 1 hr. Ki-67 reaction was visualized by using the VECTASTAIN ABC system (Vector), with diaminobenzidine as the chromogen. Sections were counterstained with hematoxylin. As the negative control, the primary antibody was replaced with a non-immune IgG at the same concentration, and no reactivity was detected. The IHC images were analyzed by using the ImmunoRatio online analysis tools [23].

### Statistical Analysis

Mean activities were calculated from at least three independent experiments performed in triplicate. The Student's two-tailed *t* test assuming unequal variance was used to determine significant differences between two groups.

## Results

### MSA synergistically enhances the growth-inhibitory efficacy of paclitaxel in MDA-MB-231 cells

**Figure 1:**
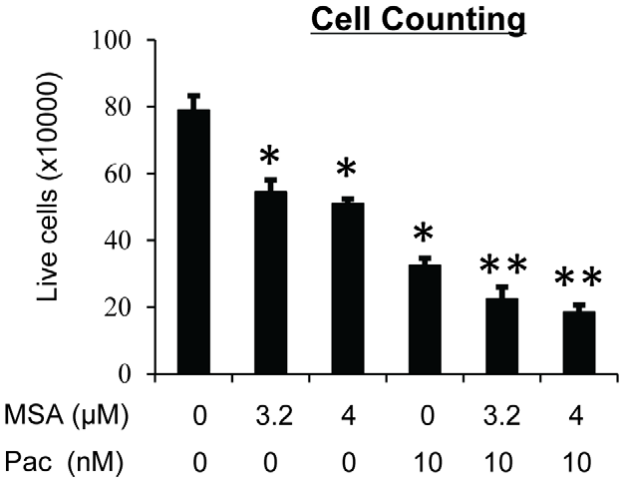
MSA enhances the efficacy of paclitaxel in inhibiting the growth of MDA-MB-231 cells. Cells were treated with 3.2 or 4 µM MSA, 10 nM paclitaxel, or the combinations for 72 hr. Live cells were counted with trypan blue staining.

We chose the most commonly-used and well-studied TNBC cell line, MDA-MB-231 [24]–[28], as the primary model for investigating the combinatory effect of MSA and paclitaxel. MDA-MB-231 was derived from pleural effusion of a breast cancer from a Caucasian patient [29]. Cells were treated with pharmacologically achievable concentrations of MSA, paclitaxel, or the two drugs in combination for 72 hr. Cell viability was first determined by the SRB assay. The cell viability in all combination groups was lower than that in the respective single-agent treatment groups (Table 1). To determine whether these combinatory effects were synergistic, the data were analyzed with the Calcusyn software (Biosoft), which calculates combination index (CI) values using the median-effect principle [30] to delineate the interaction between two drugs. A CI value of <1, 1, or >1 denotes synergism, additivity, or antagonism, respectively. All the combinations, except 2.5 µM MSA and 10 nM paclitaxel, produced a CI value of less than 1, suggesting a synergy between MSA and paclitaxel in inhibition of cell growth (Table 2). We also assessed cell viability by direct cell counting with trypan blue staining, and the data (Fig. 1) are congruent with the SRB results.

**Table 1:** Effect of MSA and/or paclitaxel on cell viability*.

Paclitaxel (nM)	MSA (µM)			
	0	2.5	3.2	4
0	100	90.0±13.2	80.3±13.2	60.7±10.5
10	47.8±5.7	44.7±0.8	35.3±1.3	29.7±1.7
20	42.6±2.8	35.8±2.3	30.3±1.7	26.1±3.8
40	38.7±2.0	37.7±3.3	28.1±0.4	25.0±2.6

* Results are expressed as % of vehicle control (mean ± SEM).

**Table 2:** Combination Index values for MSA and paclitaxel.

Paclitaxel (nM)	MSA (µM)		
	2.5	3.2	4
10	1.163	0.752	0.761
20	0.796	0.696	0.73
40	0.812	0.726	0.758

### Inhibition of cell proliferation by MSA and paclitaxel

**Figure 2:**
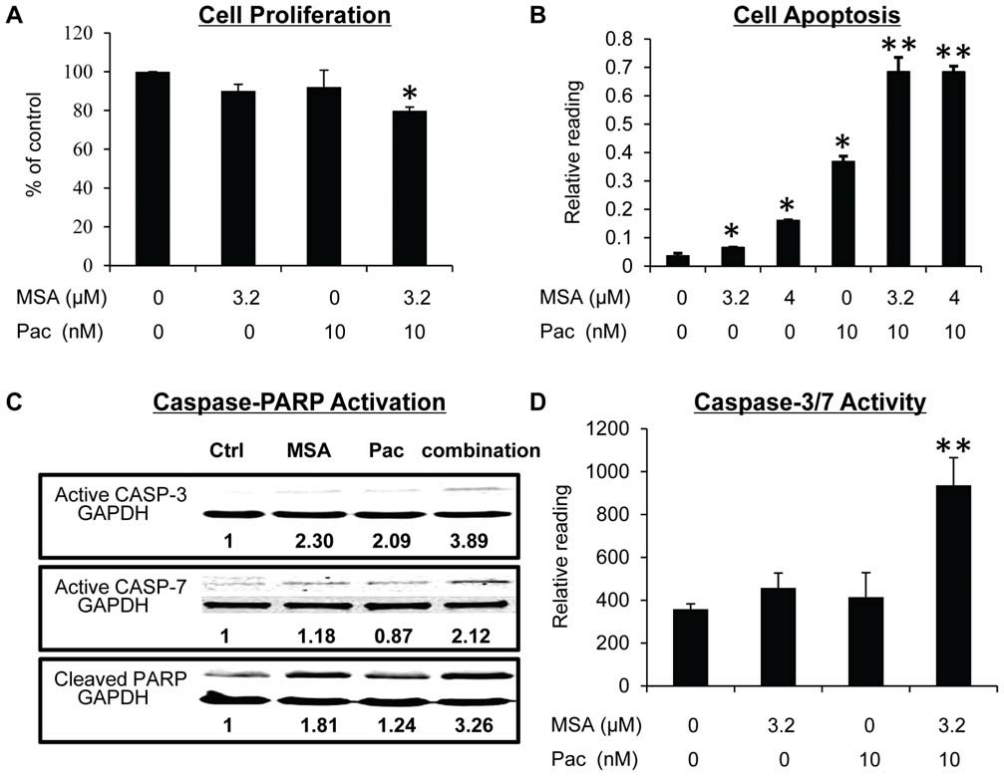
MSA enhances the efficacy of paclitaxel in inhibiting cell proliferation and inducing apoptosis, caspase activation, and PARP cleavage in MDA-MB-231 cells. Cells were treated with 3.2 µM MSA, 10 nM paclitaxel, or the combination for 16 hr. Cells were then analyzed for proliferation (**A**), apoptosis (**B**), the levels of active form of caspase-3 and -7, and cleaved PARP by Western blotting (**C**), or Caspase-3/7 activity (**D**). The BrdU, apoptosis, and caspase-3/7 activity readings were normalized by the SRB reading in cells set up in parallel. Error bars, SEM. *, statistically significant (*P*<0.05) from the control group. **, statistically significant (*P*<0.05) from both single–agent-treated samples and the control sample. Pac, paclitaxel.

To elucidate the mechanism underlying the synergism between MSA and paclitaxel, we evaluated cell proliferation in response to the treatments in MDA-MB-231 cells. Cells were treated for 16 hr at the lowest dose combination, viz., 3.2 µM MSA and 10 nM paclitaxel, which produced a synergistic effect on growth inhibition. The cell proliferation data (Fig. 2A) were normalized by the SRB reading, and expressed as percentage of the vehicle control. The combination treatment induced slight, but significant, inhibition of proliferation comparing to the control group. Similar pattern of inhibition was observed at the 24 and 36 hr time points (data not shown). The data suggest that inhibition of proliferation contributes to the synergistic effect between MSA and paclitaxel, but may not be the major contributing factor.

### MSA enhances paclitaxel-induced G2/M arrest

We next assessed the effect of the combination treatment on cell cycle distribution. The experiment was performed at the 24-hr time point in MDA-MB-231 cells. As depicted in Table 3, paclitaxel decreased the proportion of cells in G0/G1 and arrested cells in G2/M phase. At the 3.2 µM concentration, MSA alone didn't affect cell cycle distribution. However, it enhanced the paclitaxel effect by further decreasing the fraction of cells in G0/G1 phase and increasing G2/M arrest.

**Table 3:** Effect of MSA and/or paclitaxel on cell cycle distribution#.

Treatment Group	G0/G1	S	G2/M
Control	58.3±4.5	27.7±2.8	14.0±1.7
MSA	59.1±4.9	24.2±4.1	16.7±0.9
paclitaxel	33.5±5.6*	29.9±0.4	36.7±5.2*
combination	26.4±6.5**	27.3±4.4	46.3±10.9**

# Results are expressed as mean % of cells in a specific phase of cell cycle ± SEM.

* Significantly different compared to the control group (*P*<0.05).

** Significantly different compared to both single-agent-treated and control groups (*P*<0.05).

### MSA enhances paclitaxel-induced apoptosis

**Figure 3:**
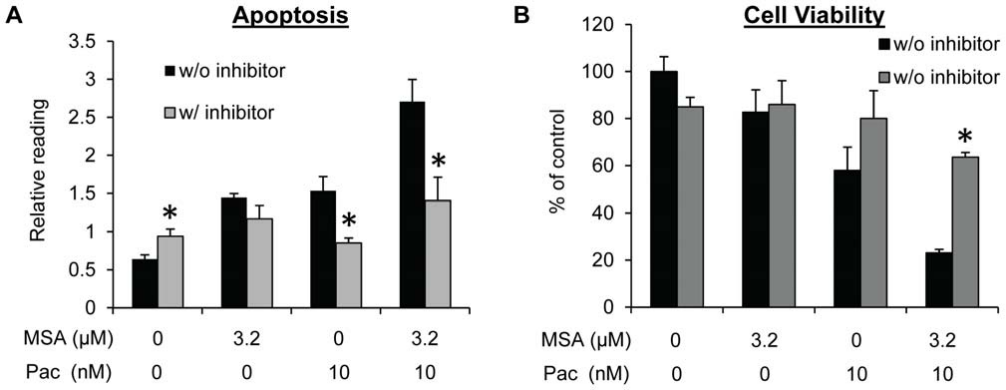
Inhibiting caspase activity attenuates apoptosis induction and growth suppression by MSA and paclitaxel in MDA-MB-231 cells. Cells were pre-treated with 50 µM pan-caspase inhibitor Z-VAD-FMK prior to treatment with 3.2 µM MSA, 10 nM paclitaxel, or the combination. Apoptosis was determined at the 16-hr time point by Cell Death ELISA (**A**), and cell viability at 72 hr by SRB (**B**). Error bars, SEM. *, statistically significant (*P*<0.05) from the corresponding control sample not treated with the caspase inhibitor. Pac, paclitaxel.

G2/M arrest induced by paclitaxel in cancer cells generally leads to extensive apoptosis [31]. We therefore evaluated apoptosis in MDA-MB-231 cells. As shown in Fig. 2B, an increase in apoptosis was observed after treatment of cells with either MSA or paclitaxel, and the increase was more pronounced as a result of the combination treatment. Caspase-PARP activation is the major pathway involved in apoptosis. We measured caspase-3 and -7 activation and PARP cleavage in MDA-MB-231 cells by Western blotting. Consistent with the apoptosis data, the combination treatment caused an enhanced activation of caspase-3 and -7 and PARP cleavage comparing to either MSA or paclitaxel alone (Fig. 2C). We also performed caspase-3/7 activity assay, and the activity data correlated well with the Western result (Fig. 2D). In addition, pre-treatment of cells with a pan-caspase inhibitor (Z-VAD-FMK) almost completely blocked apoptosis induction (Fig. 3A) and inhibition of cell viability (Fig. 3B) by paclitaxel, alone or in combination with MSA, demonstrating the caspase-PARP pathway as the major pathway involved.

### MSA enhances paclitaxel-induced inhibition of proliferation and increase of apoptosis in other TNBC cell models

**Figure 4:**
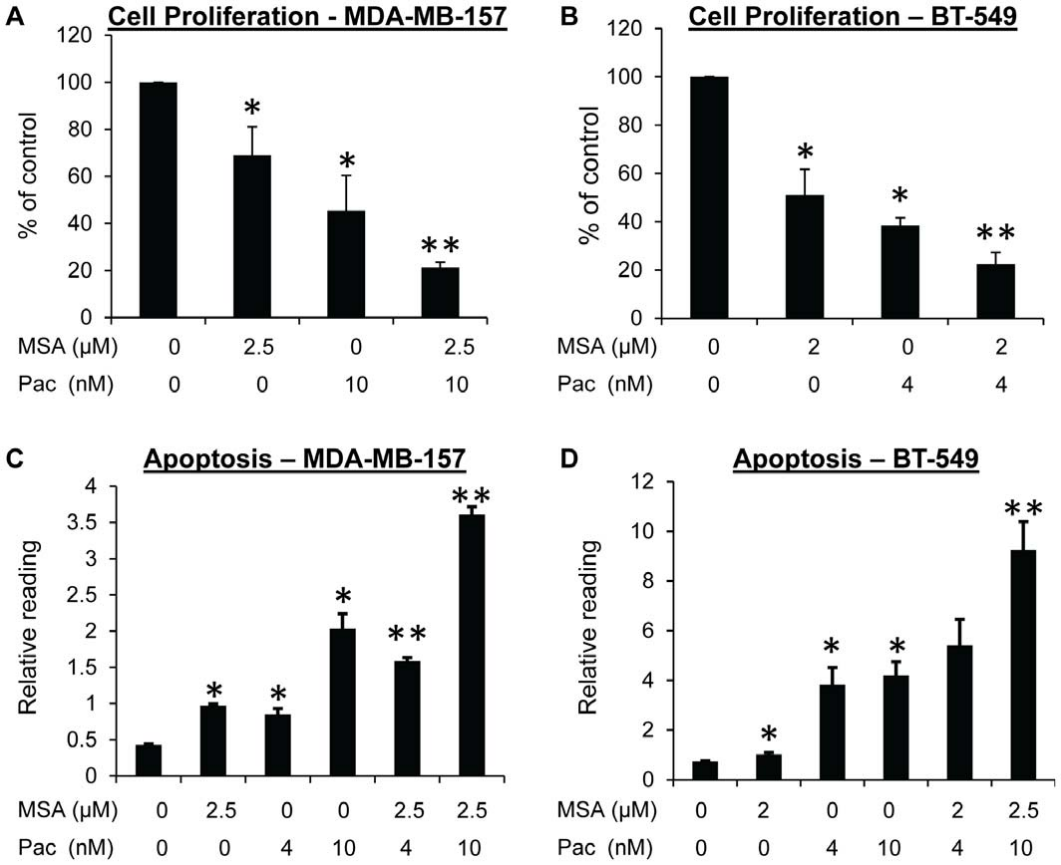
MSA enhances the effect of paclitaxel in inhibiting cell proliferation and inducing apoptosis in MDA-MB-157 and BT-549 cells. Cells were treated with MSA, paclitaxel, or the combination for 48 hr, and then subjected to analysis for proliferation (**A, B**) or apoptosis (**C, D**). The BrdU and apoptosis readings were normalized by the SRB reading in cells set up in parallel. Error bars, SEM. *, statistically significant (*P*<0.05) from the control group. **, statistically significant (*P*<0.05) from both single-agent-treated sample and the control sample. Pac, paclitaxel.

To further demonstrate the universality of the combinatory effects, we determined cell proliferation and apoptosis in two other TNBC cell models, MDA-MB-157 and BT-549. MDA-MB-157 was derived from pleural effusion of a breast cancer from an African-American patient, and BT-549 from a primary breast cancer from a Caucasian patient [29]. In both models, the combination treatments led to a more robust induction of apoptosis and inhibition of proliferation than treatment with MSA or paclitaxel alone (Fig. 4).

### MSA enhances paclitaxel efficacy in vivo

**Figure 5:**
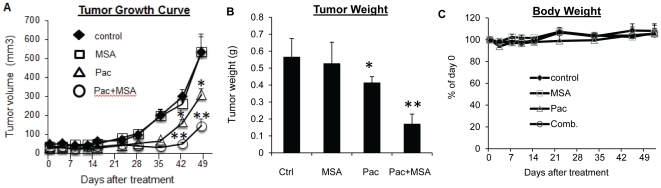
MSA enhances paclitaxel efficacy in vivo. (**A**) Tumor growth curve. Data are presented as tumor volumes in each group (n = 6 tumors/group). (**B**) Average tumor weight in each group at the end of the experiment. (**C**) Average body weight of the mice in each group. Error bars, SEM. *, statistically significant (*P*<0.05) from the control group. **, statistically significant (*P*<0.05) from both single-agent-treated sample and the control sample. Pac, paclitaxel.

**Figure 6:**
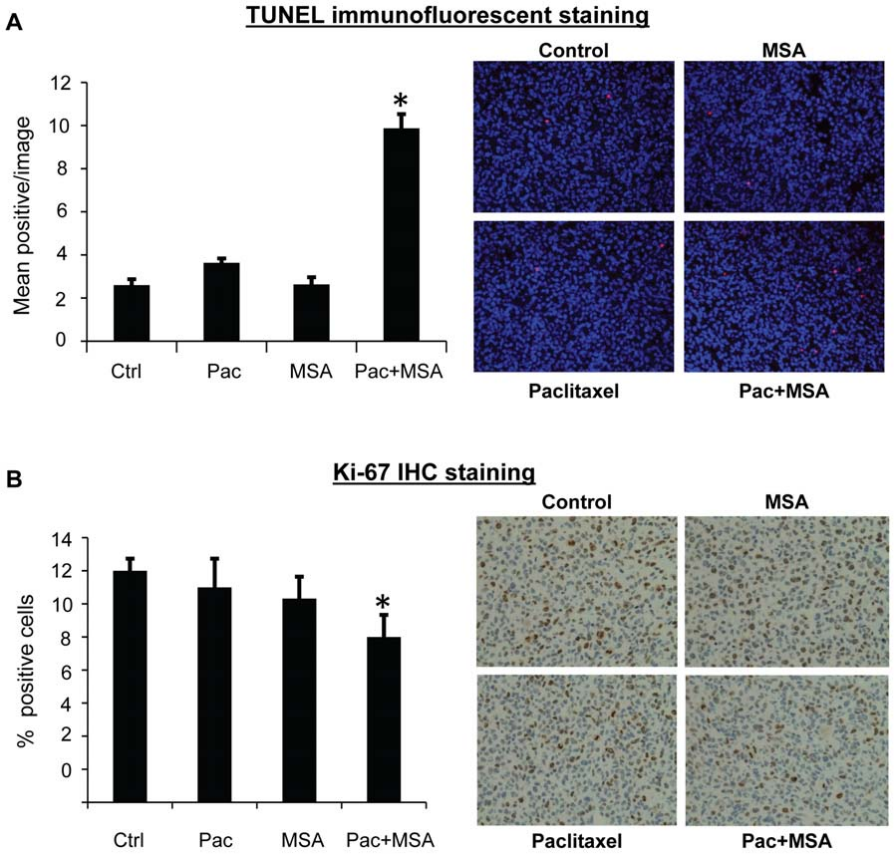
Induction of apoptosis and inhibition of tumor proliferation by paclitaxel and MSA in MDA-MB-231 xenograft. (**A**) TUNEL staining. Data are presented as mean number of TUNEL-positive cells in each image. (**B**) Ki-67 IHC. Data are presented as mean % of Ki-67-positive cells. Error bars, SEM. *, statistically significant (*P*<0.05) from both single-agent-treated sample and the control sample. Pac, paclitaxel. Right panels: Representative images from each group.

To evaluate the *in vivo* efficacy of MSA and paclitaxel, female SCID-hairless mice implanted with MDA-MB-231 cells were divided into four groups receiving saline solution as control, 3 mg selenium in the form of MSA per kg body weight daily, 10 mg/kg paclitaxel weekly, or the two drugs in combination. To be more consistent with the patient treatment schedule, all treatments were stopped after 4 doses of paclitaxel, and mice were maintained for an additional 4 weeks. As shown in Fig. 5A, MSA alone had no effect on tumor growth. In the paclitaxel group, tumor growth was suppressed until two weeks after termination of treatment, and tumor re-growth was observed on Day 42. The re-growth was greatly inhibited by the combination treatment. At the conclusion of the study, the average tumor weight in the paclitaxel group was significantly lower than that in the control group, and the combination treatment caused a more pronounced reduction of tumor weight (Fig. 5B). The combination treatment did not appear to cause more toxicity than paclitaxel alone since mice in the two groups had similar body weights (Fig. 5C). We then measured apoptosis induction by using the fluorescent TUNEL assay and proliferation index by Ki-67 IHC in the xenograft tumors. As shown in Fig. 6, the combination treatment led to a significant induction of apoptosis and inhibition of cell proliferation in the tumors.

## Discussion

In the present study, we evaluated the efficacy of MSA in combination with paclitaxel for the treatment of TNBC. Our data showed that MSA could significantly enhance the *in vitro* and *in vivo* antitumor efficacy of paclitaxel in TNBC cells. Through CI value calculation for MSA and paclitaxel, each at three different concentrations, we demonstrated that the combinatory effect on cell growth, except that of the lowest dose combination, was synergistic. The synergism was attributable to more pronounced induction of caspase-mediated apoptosis, arrest of cell cycle progression at the G2/M checkpoint, and inhibition of cell proliferation. Our *in vivo* study also showed the safety of using MSA in the combination regime. The current study is the first to show the *in vivo* effectiveness of MSA in enhancing chemotherapeutic efficacy in breast cancer. The focus on TNBC could be of more clinical importance considering the limited availability of effective treatment options for this deadly disease. An interesting observation that needs to be pointed out is that when MSA was combined with paclitaxel at their respective lowest tested concentration, the combination produced antagonistic effect (CI = 1.163). The clinical relevance of this observation requires further *in vivo* testing. If proven to be true, the information could be helpful in selecting the effective doses of the two drugs for future clinical trials.

MSA, at the dose used in our *in vivo* study, has been shown to inhibit the growth of prostate tumor xenografts [14]. However, no growth inhibition was observed in our study with the MDA-MB-231 xenograft model. This discrepancy could be due to the difference in the model system. It could also result from the difference in the treatment protocols. In the prostate tumor studies, MSA treatment was initiated right after cell inoculation and continued until the end of the experiments. However, in our study, MSA supplementation was not started until the tumors became palpable. The mice were administered MSA for only 4 weeks, and were maintained drug-free thereafter. Although there was a slight trend of growth inhibition during MSA treatment, this inhibition was not significant because the tumors were rather small. Whether MSA treatment, in a long term or at a higher dose, could inhibit the growth of TNBC tumors awaits to be determined. In our study, paclitaxel alone dramatically inhibited the growth of the xenograft tumors. However, this was not accompanied by a significant change in apoptosis or proliferation. This could again arise from our treatment and recovery experimental protocol.

Previously, we reported that MSA can induce FADD expression in MCF-7 breast cancer cells [16]. This boost of FADD expression leads to recruitment of caspase-8, resulting in a robust apoptosis induction when combined with chemotherapeutic reagents. FADD has been shown to play an important role in G2/M arrest and cell growth suppression induced by paclitaxel in breast cancer cells [32], [33]. These findings suggest that increasing FADD expression by MSA may lower the threshold for G2/M arrest induced by paclitaxel treatment, consequently leading to increased sensitivity to paclitaxel. FADD activation also may result in activation of caspase-3 in TNBC cells, which in turn leads to activation of caspase-8 or -9 and apoptosis. The FADD-caspase-3 axis should be tested in future studies to further elucidate the mechanism underlying the combinatory effect of MSA and paclitaxel.

When compared with other types of breast cancer, TNBC has an increased rate of pathologic complete response to chemotherapy. However, TNBC is associated with more prevalent and earlier-onset recurrence and shorter overall survival [2]. Failure of G2/M arrest, as a result of mitotic slippage, abnormal regulation of G2/M transition genes, polyploidy induction, or multi-nucleation, has been shown to be the major mechanism underlying acquired resistance to paclitaxel [34]. Our finding that MSA could enhance paclitaxel-mediated G2/M arrest suggests the potential of using MSA to overcome paclitaxel resistance. In fact, we generated a paclitaxel-resistant MDA-MB-231 subline by long-term treatment of MDA-MB-231 cells with low doses of paclitaxel. We found that MSA could reverse the resistance of the cells to paclitaxel (data not shown). This further demonstrates the benefit of using MSA in combination with paclitaxel for the management of TNBC.

According to the 2010 cancer statistics report from the American Cancer Society [35], cancer deaths have declined for both Caucasian and African Americans living in the United States. However, African Americans continue to suffer the greatest burden for each of the most common types of cancer. American Caucasian women have the highest incidence rate for breast cancer, although African American women are most likely to die from the disease [35]. The disparity could be attributable to many factors, such as genetic, socioeconomic, diet, and culture differences [3]. TNBC is the only subtype of breast cancer that displays racial disparity [2]. Selenium concentration has been shown to be inversely associated with the incidence of a number of cancers, including breast cancer [36], [37]. Serum selenium concentration in African Americans were found to be significantly lower than that in Caucasians [38]. It is therefore possible that the clinical benefit of MSA could be more evident in African Americans with TNBC.
